# Physical and Functional Clinical Profile of Older Adults in Specialized Geriatric Rehabilitation Care Services in Saguenay-Québec: A Retrospective Study at La Baie Hospital

**DOI:** 10.3390/ijerph19169994

**Published:** 2022-08-13

**Authors:** Maria do Carmo Correia de Lima, Mathieu Dallaire, Catherine Tremblay, Alexis Nicole, Émilie Fortin, Isabela Calixto Maluf, Josée Nepton, Anne-France Severn, Patrice Tremblay, Sharlène Côté, Julie Bouchard, Rubens A. da Silva

**Affiliations:** 1 Centre Intersectoriel en Santé Durable, Laboratoire de Recherche BioNR, Département des Sciences de la Santé, Université du Québec à Chicoutimi (UQAC), Saguenay, QC G7H 2B1, Canada; 2 Doctoral Neuropsychology Program, Université du Québec à Chicoutimi (UQAC), Saguenay, QC G7H 2B1, Canada; 3 Physical Therapy Program, Université du Québec à Chicoutimi (UQAC), Saguenay, QC G7H 2B1, Canada; 4 Medical Clinical Residence, São Paulo Hospital, Universidade Federal de São Paulo (UNIFESP), São Paulo 04024-002, Brazil; 5 Centre Intégré de Santé et Services Sociaux du Saguenay-Lac-Saint-Jean (CIUSSS SLSJ), Specialized Geriatrics Services-Hôpital de La Baie, Saguenay, QC G7H 7K9, Canada; 6 Doctoral and Master Programs in Human Movement and Rehabilitation, Universidade Evangélica de Goiás, Anapolis 75083-515, Brazil

**Keywords:** aging, older people, neuromusculoskeletal disorder, falls, rehabilitation

## Abstract

Musculoskeletal disorders, cardiovascular and neurological diseases were the most commonly debilitating conditions and risk factors associated with pain, mobility limitations, increased risk of falls and disability. Studies barely address the profile of older adults in care within a specialized geriatric rehabilitation service (SGRS) to provide subsidies for new actions within the public healthcare to reduce falls and improve management in health investments. This study aimed to establish a clinical physical and functional profile of the patients with neuromusculoskeletal and cognitive disorders and fallers in interventions within SGRS. From a retrospective study design, 127 medical records were compiled and analyzed to determine the physical and functional profile of older adults and differences according to sex, age groups and the benefits for local physical therapy intervention. The users were between 76 and 85 years of age, with diverse clinical diagnoses and debilitating conditions and impairments. A higher proportion presented gait and balance impairments and had two or more falls in 12 months. A significant effect for advanced age was observed. Overall, real benefits were reported with intervention for functional improvement, although the absence of a control group. These results have direct implications for a better understanding of a local SGRS and provide subsidies for developing new approaches for the assessment and treatment of older adults with high a risk of falls in order to reduce costs for the public health system.

## 1. Introduction

With aging of the population, there is a higher incidence of neuromusculoskeletal disorders, functional limitations and, more specifically, an increase in the number of years spent in disability, which compromises the health and quality of life of people over 65 years [1,2,3,4]. Studies have demonstrated that musculoskeletal disorders, followed by cardiovascular and neurological diseases, were the most commonly debilitating conditions and risk factors associated with pain, mobility limitations, an increased risk of falls and fractures, loss of independence and impaired ability or disability to perform activities in daily life [1,2,5,6,7].

From 2016 to 2021, the number of persons aged 65 and older in Canada rose by 18.3% to 7.0 million [8]. This is the second largest increase in 75 years, after the increase observed from 2011 to 2016 (+20.0%) [8]. In the country’s four most populated provinces, Québec has the highest proportion of people 65 years and older (20.6%, about one person in five) [8]. In Saguenay-Lac-Saint-Jean, the number of people aged 65 and older is 68,485 (24.9%) [8,9], which is higher when compared to the total population of the province.

Mobility limitations increase with age, affecting 35% of people aged 70, and the majority over 85 years old, and have been associated with increased fall risk, hospitalization, decreased quality of life and even increased mortality [1,9,10]. The determinants of mobility include several domains, ranging from physical, cognitive, psychological, environmental and financial [1,7,10,11,12].

In accordance with the Global Burden Disease (GBD) [13], with a rapidly aging global population, the demands on healthcare services to deal with disabling outcomes will require development and investment in research to identify new, more effective intervention strategies to help decision-makers identify the strategies of promotion, prevention and disease control to improve conditions for the years lived with disability and reduce healthcare costs. The scenario presented above highlights the importance of considering people aged 65 and over as a population to be studied from a sustainable health perspective, considering the impact of neuromusculoskeletal and cognitive disorders and adverse outcomes, such as functional decline, risk of falls, fractures, hospitalizations and increase in healthcare costs [1,2,5,7,13]. Based on this context, our research team developed the Aging Living-Lab Sag in a specialized public service in geriatrics rehabilitation for the first time in Saguenay-Lac-Saint-Jean region, Québec, Canada.

The Aging Living-Lab Sag is an interdisciplinary and innovation research program in the field of geriatric functional assessment/intervention and fall prevention environment, including older people with different neuromusculoskeletal disorders and neurocognitive problems on a specialized geriatric service care in rehabilitation. This research program aims to contribute to the infrastructure through the installation of various equipment (such as force platform, electromyography: EMG, kinematic and gait measurement, etc.) for rehabilitation measurements to be carried out with a clientele already present in the two geriatric services targeted: The Geriatric Day Hospital (GDH) and the Geriatric Intensive Functional Rehabilitation Unit (GIFRU) at La Baie Hospital, QC. The two settings have a multidisciplinary and interdisciplinary team, such as occupational therapists, nurses, physicians, neuropsychologists, nutritionists, speech-language pathologists, physiotherapists, social workers and geriatrics specialists.

The GDH is more related to second line outpatient evaluation and rehabilitation services for geriatric clients, including an interdisciplinary approach and individualized intervention plan. This setting contributes to complementary interventions on the front line. On the other hand, the GIFRU is a 19-bed unit for geriatric patients with intensive rehabilitation needs following a stroke or other neurological conditions and complex orthopedic conditions for which current rehabilitation services are insufficient. Again, this setting works in an interdisciplinary approach and an individualized intervention plan. It is, so far, a service integrated into the continuum of age care, particularly in the post-stroke trajectory for local population.

Summarizing, the Aging Living-Lab Sag is guided by a program that aims to implement a broad gerontology assessment model to identify fall risk factors and develop a multi-component protocol to improve physical and functional capacity and autonomy in older adults with neuromusculoskeletal and cognitive disorders for local older population at the region. The program’s mission, from a state-of-the-art principle, is related to assessment and rehabilitation intervention using high tech equipment at a local geriatric hospital for clinical care. The program was developed with the ultimate goal of reducing healthcare costs and improving quality of life and mobility for each individual tested. This program includes researchers, managers, administrative professionals, clinicians, older adults from the community and students mainly from the Université du Québec à Chicoutimi (UQAC) and collaborating with other Universities (e.g., Université de Sherbrooke), international partnerships (e.g., Florida International University, FIU USA) and the direct collaboration of CIUSSS Saguenay-Lac St-Jean at Hôpital de La Baie. Recently, there has been a significant collaboration with organizations, such as Parkinson Saguenay, MOVE50+ of Sercovie inc., Parkinson Québec, and the Le Go pour bouger!—this last is a program produced in collaboration with the Direction régionale de santé publique du CIUSSS du Centre-Sud-de-l’Île-de-Montréal de-l’Île-de-Montréal, by MOVE 50+ and the geriatric department of the Centre hospitalier Hospitalier de l’Université de Montréal. The legal use and distribution of content from these programs based in exercise practice for an older and aging group were approved for our research team when necessary to use for people. Overall, our research program also has the mission to put the patient at the center of the project as a co-participant in decisions, challenges and solutions.

As few data are available in the literature to analyze older adults with neuromusculoskeletal disorders when in the charge of a specialized rehabilitation service as happens in Quebec, the first part of our research program aimed thus to establish a clinical physical and functional profile of the patients managed in rehabilitation/physiotherapy at the specialized geriatric service at La Baie Hospital, including patients with neuromusculoskeletal and cognitive disorders and fallers. Given the nature of this investigation and the retrospective design, no specific hypothesis was established here to define this overview in a specialized geriatric rehabilitation service in Saguenay Lac-Saint-Jean.

## 2. Materials and Methods

The retrospective study was conducted at the Hôpital de La Baie (La Baie Hospital) in the GDH and GIFRU at the CIUSSS-SLSJ. The study was part of a large research program entitled: Development of a new clinical approach in rehabilitation for the management of older people with a neuro-musculoskeletal disorder and a risk of falls: A partnership project between the UQAC and the Specialized Geriatric services of the CIUSSS Saguenay-Lac-St-Jean since 2019. This research program enrolled in four different phases was approved by the local ethics committee (CER #2019 008, CIUSSS SLSJ) for the next 5 to 10 years. The procedure for this first phase, including a retrospective analysis, was to obtain an anonymous list from managers of hospital services. The participants’ files were selected confidentially and randomly by a person in charge of the archives department of the Hôpital de La Baie from patients who were admitted to the GDH or GIFRU services from 2018 to 2020. The inclusion criteria were: (1) Receiving rehabilitation treatment and (2) being 65 years of age or older. The selection of files was based on the gender and age of the participants to obtain equal representation of the sexes (50% women and 50% men) as well as proportional representation of the age groups.

This list was then sent to the archives staff who forwarded it to the two independent researchers of this program to validate the procedure. The data were pre-analyzed with the patient indication list to avoid a double-counting from the potential patients registered at different intervention sites (GDH and GIFRU) at the same time. We observed that five patients had participated in both in intensive and functional therapies (GIFRU) and in the day hospital (GDH), but the measurements were different from one place to another and did not compromise the final data analysis or any comparison performed. The medical confidential records were consulted and extracted. The clinical data from rehabilitation services were compiled in tabular form in Excel software by the researchers to include all relevant variables related to this first study. Date validation was completed by a third researcher to assure the next final step for statistical analysis.

Thus, for the 127 medical records compiled, the demographic, anthropometric and clinical measures were included (diseases and/or dysfunctions associated in the admission, limitations and disabilities). To better understand the real impairments, we used the concept (and not the code) of International Classification of Functioning, Disability and Health—ICF [14] to classify the patients into different categories of disabilities and limitations based on their diagnostics. The code of ICF was not reported here nor in the medical records of the patients. The number of falls of patients was also evaluated, as well as their performance in physical and functional capacity tests (Visual Analog Scale: VAS; Mini-Mental State Examination: MMSE [15]; Montreal Cognitive Assessment: MoCA [16]; Timed Up and Go: TUG [17]; 5 Times Sit to Stand: 5TSTS [18]; 6-Minute Walk Test: 6 MWT [19]; 10-Meter Walk Test: 10 mWT [20,21]; Berg Balance Scale: BBS [22]) when applied by the physiotherapists at the GDH and GIFRU units during rehabilitation involving traditional exercises performed in a conventional way of physical therapy management at hospital (e.g., mobility, strength and endurance exercises under sub-maximal efforts when necessary, balance and coordination and motor control). The description for prescription of these tests and questionnaires applied by clinicians are well related in the literature as cited here.

In this study, we identified the reasons for consultations, the patients who were hospitalized and those who were seen in rehabilitation for a neuromusculoskeletal disorder or after a fall event, especially for physical therapy care. Other clinical variables, such as cognitive status or neurocognitive impairments were also addressed. In fact, absolute and relative frequency of diseases and/or dysfunctions associated with the neuromusculoskeletal disorders, debilitating conditions, functional limitations, the number of falls, the length of hospitalizations and/or the number of physiotherapies during the treatment were characterized.

Descriptive analysis using mean, frequency and standard deviation values was used to describe the study sample. The data normality was tested and confirmed by the Shapiro–Wilk test when necessary. To assess the difference between men and women, an independent Student’s *t*-test with a significance level of 0.05 was performed for each demographic variable (age, weight, height, BMI, education level, length of hospitalizations, frequency of treatment and number of weeks of treatment received). To evaluate the difference between men and women for each age category (65–75 years; 76–85 years; 86+ years) at the different functional tests, a 2-factor ANOVA was performed. Those comparisons were made only for initial test scores (before rehabilitation or baseline data). An analysis was carried out between the different age groups, sexes and intervention effects on main functional capacity tests in the participants. Paired Student’s *t*-tests were performed to assess the differences between pre-intervention and post-intervention scores from a one-time factor, without the use of a control-group. Finally, effect size and % clinical differences from means were used to better determine the effect or the changes from intervention. All statistical analyses were performed with the computer package SPSS version 27 for Windows software (IBM, Armonk, NY, USA) by using 0.05 as the level of statistical significance.

## 3. Results

The sample of participants (*n* = 127) included 63 men and 64 women from GDH and GIFRU with a mean age of 80.11 ± 7.52 years. Within the sample, there was a larger proportion of patients between 76 and 85 years of age, representing 47.2%. The characteristics of the participants are shown in Table 1, comparing men and women. Significant differences were observed in weight (*p* = 0.013) as well as in height (*p* < 0.001) between the sexes. For the other variables presented, there is no significant difference between men and women.

In general, the patients received three sessions of physiotherapy per week for a total of 6 weeks of therapy before discharge; while 53 days were reported on average to define the length of hospitalization in specialized geriatric services (Table 1).

The diseases and/or dysfunctions upon admission, debilitating conditions, functional limitations and number of falls over 12 months were reported in Table 2. The diseases and dysfunctions upon admission were classified for each specialized geriatric service (GDH and GIFRU) at the physical therapy services in this context of analysis. At the GDH, 18% presented neurocognitive disorders, 17% presented with a diagnosis of cerebral vascular accident (stroke), 14% with Parkinson’s disease (PD), 5% presented deconditioning/loss of autonomy and 4% were admitted for fractures. Twenty-four percent (24%) were classified as “others” (combination of different comorbidities).

In the GIFRU services, a higher proportion of individuals consulted for stroke (29%), 20% with fractures, 10% presented deconditioning/loss of autonomy, 4% with PD, 1% presented severe and complex neurocognitive disorders and 19% were classified as “others”.

Based on ICF [14], we observed the physical deficiencies of patients in both services (GDH and GIFRU). A total of 24% of patients presented significant muscle weakness, followed by cardiovascular (17%) and muscular (13%) deconditioning and chronic pain (13%). The two main functional activity limitations, representing 75% of the sample, were related to gait (39%) and balance (36%) deficits (subjective and objective analysis by physical tests), which indicates a significant risk of falls among all these patients. The number of falls over 12 months was 42%, reporting two or more fall events at the time (Table 2).

As for the functional tests and questionnaires completed by the users in both geriatric services (GDH and GIFRU), most of the results do not show any significant differences between men and women, as presented in the tables below (Table 3). No significant differences between sexes were found for pain measurements from VAS, cognitive status quantified by MMSE [15] and MoCA [16] scores and balance measurements from BBS [22] (*p* > 0.05) (Table 3). However, one of the functional tests showed a significant difference between the sexes, the 10 mWT at usual and maximum walking speed [20,21] (Table 3). For these tests, men reported a mean of 0.74 m/s (usual) and 1.11 m/s (maximum), while women were lower at 0.57 m/s (usual) and 0.75 m/s (maximum), respectively, with a clinical difference of 23% (*p* = 0.023; in usual) and 32% (*p* = 0.002; in maximum) between groups for each speed.

At the time of analysis, a significant age effect was observed across the different variables. The individuals were classified into three age groups: (1) 65–75 years, (2) 76–85 years and (3) 86 years and older. As expected, aging negatively impacts performance on functional tests (Table 4 and Table 5), where the older the individual, the poorer the physical or functional performance. A decline with advancing age was clearly observed during the performance from the TUG [17], the 10 mWT at usual and maximum speeds [20,21], as well as for the balance on the BBS [22] (Table 4 and Table 5).

In the results from Table 5, significant changes were reported between the 65–75 and 86+ groups and between the 76–85 and 86+ groups for functional tests. However, no significant changes were observed between the 65–75 and 76–85 age groups. The difference between the means in TUG [17] was, for example, 2.73 s for the 65–75 group versus the 76–85 group, while the difference between the 65–75 and 86+ group was 10.94 s (Table 5).

Finally, the effect of interventions at the specialized geriatric rehabilitation units (GDH and GIFRU) based on a physiotherapy intervention including people with weak to moderate cognitive disorders was also analyzed. Overall, the types of therapy integrated more functional exercises, notably gait training, static and dynamic balance exercises and mobility. For each of the tests performed, pre-and post-intervention, a significant, or even highly significant, difference was demonstrated (*p* < 0.01) for a significance level of 0.05. These differences were related to an improvement in test performance after therapy, except for the 6 MWT [19], where there was no improvement over time (Table 6). The effect size is small for each of the results presented, despite the clinical improvements ranging from 5.2% to 15.5% across the different measurements or tests applied (Table 6).

## 4. Discussion

A This study provides a general profile of the patients from specialized geriatric services at the CIUSSS Saguenay-Lac-Saint-Jean, Hôpital de La Baie (GDH and GIFRU) between 2018–2020 for the first time. The mean age of patients was between 76 and 85 years old, with individuals presenting diverse clinical diagnoses and deficiency conditions. Muscle weakness, cardiovascular and muscle deconditioning were the most common impairments seen at the physiotherapy services. A higher proportion presented impaired gait and balance and had experienced two or more falls in 12 months. Additionally, interventions completed within the specialized services resulted in significant improvements for these users.

Several studies demonstrated that functional limitation is common in older adults and increases with age and health conditions, such as cardiopulmonary diseases, neurological conditions, diabetes mellitus, cancer, obesity, dementia, affective disorders and fractures [1,10,23,24]. In addition, the coexistence of two or more health conditions often creates more disability than would be expected [1,23,25]. In this study, the services presented a percentage of combination of comorbidities of 24% in GDH and 19% in GIFRU, and a mixture of clinical diagnoses, such as stroke, Parkinson’s, fractures and neurocognitive disorders.

In the physical domain, gait, balance and strength play an important role. Mobility limitation during aging is associated with the loss of strength and/or function and falls [1,7,12,26]. Approximately one third to one half of individuals aged 65 years or older report difficulties related to walking or climbing stairs [1,7,12,26]. According to Osaba et al. [26], the prevalence of gait and balance deficits increases with age and is associated with a higher incidence of falls. At the age of 70 years and older, the prevalence of gait disturbance is 35% [26,27]. From 65 to 69 years of age, it is estimated that 13% have balance impairment, and this proportion increases to 46% at 85 years of age or older [26].

In this study, few differences in functional tests were noted between men and women, but those related to age were significant, notably for gait and balance. This lack of significant differences between sexes can be explained by measurements from subjective or physical tests, without including high-tech equipment for postural control and walking analysis, such as a force or kinematic platform by systems often used in research (Vicon, GaitRite, cameras). In addition, the sample from two services is also heterogeneous in some ways, presenting various neuromusculoskeletal disorders with different severities between them and other associated comorbidities.

Gait has been suggested as the “sixth vital sign” in geriatrics and used as a marker of physical function and as a predictor of falls [1,28,29,30]. Studies have shown biological differences between sexes in gait and speed, men walking faster compared to women [31,32], which was attributed to the longer stride length produced by men [33,34,35]. From the biological differences related to sex, the 10 mWT showed a significant difference between men and women at usual and maximum walking speeds (Table 3). Men performed better on both tests than women. The means that men’s walking speeds were 0.74 m/s and 1.11 m/s, while women’s were 0.57 m/s and 0.75 m/s, with a clinical difference of 23% and 32%, respectively, for each walking speed (usual and maximum). According to Samson et al. [35], due to age, height and body weight, the absolute values of walking speed were lower in women than in men, at all ages. Considering body weight, the percentage of variance explained for walking speed was 37%, and for stride length 59%. Cadence showed no association with age, height and body weight [35].

However, Kasović et al. [36] reported a dependence regarding a higher body mass index in women, significantly associated with slower gait speed. Apparently, our results were not confounding by anthropometric measures, because both groups were homogenous in regard to BMI. In fact, in the present study, when comparing men and women in both services (GDH and GIFRU), no significant differences between the sexes were observed for BMI. The systematic review of Herssens et al. [30] demonstrated that aging decreased preferred walking speed, cadence, step and stride length. However, any sex-specific differences in gait changes with aging were less investigated [1,30].

Regarding the established age groups (65–75 years, 76–85 years and 86 years and older), our results suggested that advancing age negatively affects performance on functional tests. The functional decline was observed between the age groups in: (1) TUG’s time of execution (difference between the 65–75 and 86+ of −10.94 s and of −13.68 s between 76–85 versus 86+); (2) 10 mWT’s time of execution, in the usual walking speed (difference of 0.26 s between 65–75 and 76–85 versus 86+) as well as in maximum walking speed (difference of −0.26 s in 65–75 versus 76–85 and 0.36 s in 76–85 for 86+); and (3) the worst performance in BBS (the difference between the 65–75 and 86+ was 7.06 points and 9.09 points between 76–85 versus 86+). Studies show that maximum walking speed declined at an earlier age than usual walking speed [1,10,37,38]. In the InCHIANTI study [10], the usual walking speed was stable in persons up to 65–70 years, whereas the maximum walking speed started to decline after the age of 40–50 years [1,10]. In addition to advancing age, patients with neuromuscular disorders have impaired mobility, gait and balance, resulting in a slowness of gait and balance disorders and an increased risk of falls [1,2,5,7,24,26,37,38,39,40,41] for participants’ profiles in this study.

Most functional tests in this study showed improvements ranging from 5.2% to 15.5% with the intervention on the specialized geriatric rehabilitation units (GDH and GIFRU). In both units, the physiotherapists performed their interventions using the ICF approach [14,42] and utilized practice evidence, including moderate cognitive disorders, to understand the users’ care and support needs [42,43,44,45]. The results of meta-analysis studies emphasize the clinical relevance of combined cognitive and physical exercise interventions, as they may improve the cognitive deficits, mood and performance of activities of daily living [43,44,45]. However, any generalization of these results must be attempted with caution. Considering the heterogeneity of clinical diagnoses and the retrospective study design, phase 1 presents limitations to explore or determine specific cause–effect relationships or direct casual comparisons. No case–control group or clinical design was used to establish cause–effect from local interventions to support their benefits or superiority when compared to other methods.

Thus, the research program’s next steps are to evaluate the frailty criteria [46] and explore the specific parameters of gait and postural control using state-of-the-art equipment [47], such as kinematic measures using GaitRite and postural control with the use of force platforms, to implement a more precise and objective approach for mobility/balance training and retention, thus implementing a fall prevention program for the users of the specialized geriatric services of the CIUSSS Saguenay-Lac-Saint-Jean, Hôpital de La Baie (GDH and GIFRU).

Furthermore, our research team will introduce, for the first time in Saguenay, the OTAGO exercise program based on specific balance, strength and muscular endurance exercises as well as patient education on the risks of falls, which can reduce falls by 35% [48,49]. This program may be a cost-effective intervention for the healthcare system to reduce expenses associated with falls [48,50,51]. In addition, the research program incorporates the aim of establishing sustainable and interdisciplinary health perspectives for older adults, including future partnerships with the purpose of improving and maintaining well-being in old age by focusing on natural environments [52,53,54,55]. Some studies highlight that spending time in natural environments offers physical and psychological benefits, such as positive effects on well-being, fewer feelings of anxiety, reduced depressive symptoms, increased adherence to physical exercise programs and the motivation to move outdoors, which are all important facilitators for those with walking difficulties [52,53,54,55]. Our research group is currently exploring this dimension in specialized geriatric care in this region of Québec (Saguenay).

## 5. Conclusions

The patients of the specialized geriatric services presented factors that predispose them to negative health outcomes, such as the presence of neuromuscular disorders; debilitating conditions, such as muscular weakness; and cardiovascular and muscle deconditioning. In addition, this sample was characterized for gait and balance impairments and demonstrated a strong profile for fallers. No specific or systematic sex differences were observed, but a significant effect for advanced age was observed on all the variables investigated. Significant benefits of therapy were reported for the patients for physical and functional measures, without the presence of a control group. These results have direct implications for the better understanding of a specialized geriatric rehabilitation service and provide subsidies for new actions. These actions aim at developing new methods or approaches for the assessment and treatment of older adults at high risk of falls.

## Figures and Tables

**Table 1:** Characteristics of the patients from GDH and GIFRU services in Hôpital de La Baie, Saguenay, Quebec.

Variables	Men (*n* = 63) Mean (±SD)	Women (*n* = 64) Mean (±SD)	Total (*n* = 127) Mean (±SD)	*p*-Value
Age (years)	79.78 ± 6.95	80.44 ± 8.08	80.11 ± 7.52	0.623
BMI (kg/m^2^)	24.93 ± 3.47	26.62 ± 6.61	25.80 ± 5.36	0.122
Weight (kg)	70.03 ± 9.70	63.86 ± 16.02	66.81 ± 13.67	0.013 *
Height (m)	1.67 ± 0.07	1.55 ± 0.06	1.61 ± 0.09	<0.001 *
Education level (years)	10.04 ± 3.99	10.25 ± 3.51	10.06 ± 3.63	0.766
Length of hospitalizations (days)	53.22 ± 49.38	53.56 ± 41.41	53.40 ± 45.19	0.967
Number of sessions per week	3.01 ± 1.30	3.06 ± 1.14	3.04 ± 1.22	0.800
Number of weeks of therapy	6.02 ± 5.49	7.23 ± 6.80	6.63 ± 6.19	0.283

SD: Standard Deviation; BMI: Body Mass Index. * *p* < 0.05.

**Table 2:** Clinical and functional characteristics of the patients from the GDH and GIFRU services in Hôpital de La Baie, Saguenay, Quebec.

Variables	GDH (%)	GIFRU (%)
Diseases and/or dysfunctions associated upon admission		
Cerebral vascular accident	17.0	29.0
Parkinson’s disease	14.0	4.0
Neurocognitive disorders	18.0	1.0
Fracture	4.0	20.0
Deconditioning/loss of autonomy	5.0	10.0
others	24.0	19.0
	GDH + GIFRU (%)	
Debilitating conditions		
Loss of muscle strength	24.0	
Decrease in cardiorespiratory endurance	17.0	
Decrease in muscle endurance	13.0	
Presence of pain	13.0	
Fatigue	9.0	
Decreased mobility for walking	8.0	
Dyspnea	6.0	
Others	6.0	
Spasticity	4.0	
Functional limitations		
Gait	39.0	
Balance	36.0	
Use of stairs	25.0	
Numbers of falls (12 months)		
0	29.0	
1	29.0	
2 or more	42.0	

**Table 3:** Clinical and functional test results of the patients in both services (GDH and GIFRU) in Hôpital de La Baie, Saguenay, Quebec.

Variables	Men (*n* = 63) Mean (±SD)	Women (*n* = 64) Mean (±SD)	*p*-Value
VAS	4.16 (±2.77)	4.64 (±2.79)	0.771
Risk factors for falls	8.95 (±2.76)	9.24 (±2.61)	0.608
MMSE	23.82 (±4.04)	24.71 (±4.23)	0.437
MoCA	18.88 (±5.56)	19.33 (±7.27)	0.939
TUG (s)	21.23 (±16.01)	26.21 (±16.58)	0.096
5TSTS (s)	17.71 (±7.35)	20.94 (±12.52)	0.139
6 MWT (m)	267.38 (±135.30)	220.81 (±93.23)	0.337
10 mWT (usual) (m/s)	0.74 (±0.32)	0.57 (±0.27)	0.023 *
10 mWT (maximum) (m/s)	1.11 (±0.38)	0.75 (±0.36)	0.002 *
BBS (/56)	44.89 (±8.58)	40.84 (±9.28)	0.09

SD: Standard Deviation; VAS: Visual Analog Scale; MMSE: Mini-Mental State Examination; MoCA: Montreal Cognitive Assessment; TUG: Timed Up and Go; 5TSTS: 5 Times Sit to Stand; 6 MWT: 6-Minute Walk Test; 10 mWT: 10-Meter Walk Test; BBS: Berg Balance Scale. * *p* < 0.05.

**Table 4:** Functional test scores, by age group, in both services (GDH and GIFRU) in Hôpital de La Baie, Saguenay, Quebec.

Variables	65–75 Years *n* Mean (±SD)	76–85 Years *n* Mean (±SD)	86 Years and Older *n* Mean (±SD)
TUG (s)	21 21.79 (±13.97)	41 19.05 (±13.54)	24 32.74 (±19.36)
5TSTS (s)	10 20.27 (±14.08)	22 17.08 (±8.43)	11 21.94 (±7.74)
6 MWT (m)	11 285.45 (±156.33)	29 264.86 (±104.44)	10 186.70 (±113.53)
10 mWT (usual) (m/s)	17 0.79 (±0.37)	44 0.84 (±0.29)	27 0.53 (±0.25)
10 mWT (maximum) (m/s)	12 1.02 (±0.4)	37 1.03 (±0.38)	19 0.66 (±0.36)
BBS (/56)	17 44.06 (±8.57)	47 46.09 (±6.68)	31 37.00 (±10.08)

SD: Standard Deviation; TUG: Timed Up and Go; 5TSTS: 5 Times Sit to Stand; 6 MWT: 6-Minute Walk Test; 10 mWT: 10-Meter Walk Test. BBS: Berg Balance Scale.

**Table 5:** Comparison of functionality between age groups, in both services (GDH and GIFRU) in Hôpital de La Baie, Saguenay, Quebec.

Variables	Age Group	Age Group	Mean Difference (±SD)	*p*-Value
TUG (s)	65–75	76–85	2.73 (±4.11)	0.784
		86+	−10.94 (±4.58)	0.050 *
	76–85	65–75	−2.73 (±4.11)	0.784
		86+	−13.68 (±3.94)	0.002 *
	86+	65–75	10.94 (±4.58)	0.050 *
		76–85	13.68 (±3.94)	0.002 *
5TSTS (s)	65–75	76–85	3.18 (±3.60)	0.653
		86+	−1.67 (±4.13)	0.913
	76–85	65–75	−3.18 (±3.60)	0.653
		86+	−4.86 (±3.49)	0.355
	86+	65–75	1.67 (±4.13)	0.913
		76–85	4.86 (±3.49)	0.355
6 MWT (m)	65–75	76–85	−6.41 (±42.73)	0.988
		86+	71.75 (±52.73)	0.370
	76–85	65–75	6.41 (±42.73)	0.988
		86+	78.16 (±44.25)	0.193
	86+	65–75	−71.75 (±52.73)	0.370
		76–85	−78.16 (±44.25)	0.193
10 mWT (usual) (m/s)	65–75	76–85	−0.004 (±0.07)	0.998
		86+	0.26 (±0.08)	0.008 *
	76–85	65–75	0.004 (±0.07)	0.998
		86+	0.26 (±0.06)	<0.001 *
	86+	65–75	−0.26 (±0.08)	0.008 *
		76–85	−0.26 (±0.06)	<0.001 *
10 mWT (maximum) (m/s)	65–75	76–85	−0.009 (±0.11)	0.996
		86+	0.35 (±0.12)	0.018
	76–85	65–75	0.009 (±0.115)	0.996
		86+	0.36 (±0.098)	0.001 *
	86+	65–75	−0.35 (±0.128)	0.018 *
		76–85	−0.36 (±0.098)	0.001 *
BBS (/56)	65–75	76–85	−2.03 (±2.31)	0.656
		86+	7.06 (±2.46)	0.014 *
	76–85	65–75	2.03 (2.31)	0.656
		86+	9.09 (±1.89)	<0.001 *
	86+	65–75	−7.06 (±2.46)	0.014 *
		76–85	−9.09 (±1.89)	<0.001 *

SD: Standard Deviation; TUG, Timed Up and Go; 5TSTS, 5 Times Sit to Stand; 6 MWT, 6-Minute Walk Test; 10 mWT, 10-Meter Walk Test. BBS: Berg Balance Scale. * *p* < 0.05.

**Table 6:** Impact of conventional physiotherapy on the specialized geriatric rehabilitation units (GDH and GIFRU) in Hôpital de La Baie, Saguenay, Quebec.

Variables	Score Initial Mean (±SD)	Score Final Mean (±SD)	*p*-Value	Effect Size (d)	%Δ Clinical Improvement
TUG (s)	23.54 (±16.37)	19.88 (±12.31)	<0.01 *	0.26	15.5%
5TSTS (s)	19.07 (±9.84)	16.35 (±7.31)	<0.01 *	0.32	14.3%
6 MWT (m)	247.82 (±120.61)	230.44 (±122.33)	0.03 *	0.14	−7.0%
10 mWT (usual) (m/s)	0.66 (±0.31)	0.70 (±0.31)	<0.01 *	0.13	6.1%
10 mWT (maximum) (m/s)	0.92 (±0.41)	0.97 (±0.41)	<0.01 *	0.12	5.4%
BBS (/56)	42.76 (±9.14)	44.97 (±7.05)	<0.01 *	0.27	5.2%

SD: Standard Deviation; TUG: Timed Up and Go; 5TSTS: 5 Times Sit-to-Stand; 6 MWT: 6-Minute Walk Test; 10 mWT: 10-Meters Walk Test; Cohen’s d = 0.2: low, 0.5: moderate, 0.8 = high; BBS: Berg Balance Scale. * *p* < 0.05.

## Data Availability

Not applicable.
